# Relationship Between Time of Admission, Help-Seeking Behavior, and Psychiatric Outcomes: “From Dusk Till Dawn”

**DOI:** 10.3389/fpsyt.2022.842936

**Published:** 2022-04-27

**Authors:** Marius Knorr, Andreas B. Hofmann, Dimitrina Miteva, Vanessa Noboa, Katrin Rauen, Fritz Frauenfelder, Erich Seifritz, Boris B. Quednow, Stefan Vetter, Stephan T. Egger

**Affiliations:** ^1^Department of Psychiatry, Psychotherapy, and Psychosomatics, Psychiatric University Hospital of Zurich, University of Zurich, Zurich, Switzerland; ^2^Faculty of Medicine, University San Francisco de Quito, Quito, Ecuador; ^3^Department of Geriatric Psychiatry, Psychiatric University Hospital of Zurich, University of Zurich, Zurich, Switzerland; ^4^Institute for Stroke and Dementia Research, Laboratory of Experimental Stroke Research, Ludwig Maximilian University of Munich, Munich, Germany; ^5^Faculty of Medicine, Department of Psychiatry, University of Oviedo, Oviedo, Spain

**Keywords:** help-seeking behavior, day and time of admission, coercive measures, service use, psychiatric outcomes

## Abstract

**Introduction:**

Day and time of admission influence treatment outcomes and prognosis in several medical specialties; this seems related to resources' ability. It is largely unknown whether this also applies to mental health services. We investigate the relationship between time of admission, patients' demographic and clinical profile, and treatment outcomes.

**Methods:**

Demographic and clinical profiles of admitted and discharged patients to a general psychiatric ward between January 1st, 2013 and December 31st, 2020, were analyzed. In addition, we used the last year (i.e., 2020) to monitor rehospitalization. Time of admission was defined as weekdays (working day, weekend) and dayshifts (daytime, dusk, and dawn).

**Results:**

During the study period, 12,449 patient admissions occurred. The mean age of the sample was 48.05 ± 20.90 years, with 49.32% (*n* = 6,140) females. Most admissions (*n* = 10,542, 84%) occurred on working days. Two-fifths of admissions (39.7%, *n* = 4,950) were compulsory, with a higher rate outside daytime hours. Patients had slight differences in the clinical profile, resulting from evaluating the different items of the Health of Nation Outcome Scale (HoNOS). Patients admitted on night shifts, weekends, and holidays showed a shorter length of stay; patients compulsorily admitted during daytime (disregarding the day of the week) had a longer length of stay. All patient groups achieved a robust clinical improvement (i.e., an HoNOS score reduction of around 50%), with similar readmission rates.

**Discussion:**

The main finding of our study is the relationship between “daytime hours” and fewer compulsory admissions, a result of the interplay between demographics, clinical characteristics, and out-of-clinic service availability (such as ambulatory psychiatric- psychological praxis; day-clinic; home-treatment). The differing clinical profile, in turn, determines differences in treatment selection, with patients admitted after office hours experiencing a higher rate of coercive measures. The shorter length of stay for out-of-office admissions might result from the hospitalization as an intervention. These results should encourage the implementation of outpatient crisis-intervention services, available from dusk till dawn.

## Background

In several medical conditions, the day and time of admission influence treatment and, therefore, outcome and prognosis. Patients admitted on weekends for medical, surgical, and obstetric care tend to have more complications and higher mortality rates. This phenomenon is related to different health service factors, mainly resource availability (1–3). Patients admitted to psychiatric treatment on the weekend have worse outcomes and higher readmission rates (4–6). In contrast to other disciplines, the day and time of psychiatric hospitalization are related to several clinical characteristics, influencing how patients seek and access mental health services (7, 8). Admission in the hospital is a complex process; where help-seeking (or treatment-seeking) behaviors play a pivotal role, determining if someone is admitted voluntarily or involuntarily through a compulsive admission order. To further complicate things, help-seeking behavior is in itself diverse. It includes a wide range of professions, such as mental health professionals (i.e., psychiatrists, psychologists); non-mental health professionals (i.e., emergency services, general practitioners); and non-health professionals (e.g., clergy, teachers, social workers, police) (9). Beyond individual characteristics of the patients, their psychosocial circumstances, such as support from relatives, friends, and work colleagues, also seem to influence help-seeking behavior (7, 8, 10, 11). Non-mental health professionals and services are frequently overwhelmed by their patients' psychiatric problems (12, 13). Finally, the resources and organization of mental health services and legal regulations steer patient flows; as the legal threshold for involuntary hospitalization, the readiness and availability of inpatient and outpatient treatment capabilities; and the organization of psychiatric consultation-liaison services (14–17). The process and backgrounds underlying the decision to hospitalize a person for treatment are diverse; the current literature is sparse and inconsistent (18, 19). Our study aims to gain insight into the interrelationships between patient characteristics, mental health services, and legal framework. We expect this to lead to a better understanding of psychopathology and help-seeking behavior, to improve treatment and outcomes of psychiatric patients. In a sample of continuously admitted patients for acute psychiatric treatment, we explore the relation between day and time of admission with demographic and clinical profile, treatment, outcomes, and subsequent service use.

## Methods

### Study Design and Data Source

The Center for Acute Psychiatric Disorders, as part of the Psychiatric University Hospital of Zurich, is responsible for the inpatient treatment of adults aged 18–65 years in the city of Zurich and its surroundings, with a catchment area of ~500,000 habitants. It provides 100 beds distributed in eight wards to treat psychiatric crises and emergencies. Routine demographic and clinical data are recorded for each admission (and discharge). We drew on data from an 8-year period (January 1st, 2013- December 31st, 2020); the last year was solely used to observe service use after discharge. Therefore, only a patient's first admission during the observation period, with a 1-year follow-up after discharge, was included in the analysis. The Ethics Committee of the Canton of Zurich authorized the use of anonymized data for research and publication purposes (BASEC: 2018-01906).

### Day and Time of Admission, Help-Seeking Behavior, and Pathway to Admission

The day and time of admission were recorded and categorized according to the daytime and weekday. Daytime was divided into three shifts: “daytime” (i.e., office hours from 8 to 17 h), “dusk” (from 18 h to midnight), and “dawn” (from midnight to 7 h). The days of the week were categorized as: “working days” (i.e., regular weekdays from Monday to Friday) and “weekends” (i.e., Saturdays, Sundays, and public holidays).

We operationalized “help-seeking behavior” 2-fold (9, 20, 21). First, as either “voluntary” or “involuntary” (i.e., by a compulsory admission order). Compulsory admission orders in the Canton of Zurich, Switzerland, may be issued by a qualified physician if the treatment or care of a mental disorder cannot be provided otherwise; in contrast to other countries, impending danger is not essential (22). Thus, allowing sensing the discrepancy in the need for treatment between medical judgment the patients' insight. Second, we further differentiated the pathway to admission according to referral as: “emergency services” (i.e., an ambulance or the emergency department of a general hospital); “mental health professionals” (i.e., psychiatrist and for voluntarily admitted patients also clinical psychologists); “health professionals” (i.e., any other medical discipline); thus allowing us to infer that care (i.e., therapy) was not possible at these sites. In addition, we established a fourth and exclusive category, “self-referral” (i.e., own initiative; walk-in: or advice by non-medical professionals) for voluntarily admitted patients.

### Diagnoses, Outcomes, and Service Use Parameters

Attending psychiatrists, psychiatry residents, or clinical psychologists carried out diagnoses and clinical ratings. Relevant information was derived from the clinical interview and reports of nursing staff, social workers, and significant others. Diagnoses were made according to the International Classification of Diseases, 10th edition (23) criteria and then grouped in one of eight diagnostic categories: “Dementia and Neurocognitive Disorders” (F0); “Alcohol Use Disorder” (F10); “Substance Use Disorder” (F11–F19); “Schizophrenia Spectrum Disorders” (F2); “Mania and Bipolar Disorder” (F30–F31), “Major Depressive Disorder” (F32–F34); “Anxiety and Stress-related Disorders” (F4); and “Personality Disorders” (F6). In addition to the main treatment diagnosis, we addressed psychiatric comorbidity with “Alcohol or Substance Use Disorder” (F10–F19); and “Personality Disorder” (F6). We chose diagnostic categories to be representative and large enough for statistical analysis and comparison.

For clinical rating, we used the Clinical Global Impressions Scale (CGI) and the Health of the Nation Outcome Scales (HoNOS). The CGI is a brief, easy-to-use measurement tool that assesses the severity and response to treatment in different subscales (24, 25). The Severity of Illness (CGI-S) scale is rated on a seven-point Likert-like scale response format which ranges from “1” (normal) to “7” (extremely ill). The Global Improvement (CGI-I) evaluates the change (i.e., either improvement or deterioration) in comparison to a previous CGI evaluation. It is rated on a seven-point Likert-like scale that ranges from “1” (very much improved) to “7” (very much worse), whereby a score of “4” indicates no change.

The HoNOS is a measurement instrument to assess the severity and the burden of a psychiatric disorder in 12 different domains (26, 27). Each item is rated on a five-point Likert-like scale ranging from “0” (no problem) to “4” (severe to very severe problem). The domains are defined at item level: 1. “Overactive, aggressive, disruptive or agitated behavior;” 2. “Non-accidental self-injury;” 3. “Problem-drinking or drug-taking;” 4. “Cognitive problems;” 5. “Physical illness or disability problems;” 6. “Problems associated with hallucinations and delusions;” 7. “Problems with depressed mood;” 8. “Other mental and behavioral problems” (including phobic; anxiety; obsessive-compulsive; mental strain/tension; dissociative; somatoform; eating; sleep; sexual and other), 9; “Problems with relationships;” 10. “Problems with activities of daily living;” 11. “Problems with living conditions” and 12. “Problems with occupation and activities.” There is a glossary with a clear and concise definition for each domain and rating instructions. We evaluated the HoNOS at scale level (i.e., sum score ranging from 0 to 48) and item level. We considered, items rated three or four as clinically significant and part of the patients' care and treatment plan (28–30).

We extracted the pharmacological and non-pharmacological treatments prescribed and coercive measures executed during hospitalization from the electronic health record. The pharmacological treatment comprises all medication approved by the national authority. We classified the pharmacological treatment according to their indication: “antipsychotics,” “antidepressants,” “mood- stabilizers” (i.e., lithium and antiepileptics), “anxiolytics and hypnotics,” “narcotics” (i.e., scheduled medication as amphetamines, or opioids), and “other” (pharmacological treatment for non-psychiatric disorders).

We defined the non-pharmacological treatments as “crisis intervention” (short-term treatment to mental, emotional, and behavioral distress due to a psychiatric disorder), “counseling” (professional guidance, information, and motivational counseling), “observation” (of symptoms and behavior for diagnostic appraisal), “psychotherapy” (either single or group), and “occupational therapy” (art, work, sport and leisure activities). Patients were entitled to receive more than one treatment modality.

Coercive measures were classified as: “isolation- restraint,” “forced medication,” and “compulsory retention;” patients could have undergone one or more coercive measures during their hospitalization. If a patient had been compulsorily admitted, we also recorded those that changed their willingness and remained voluntarily in psychiatric treatment.

We defined the type of discharge as either “regular;” “irregular” (discharge against medical advice, court order, or patients who leave the hospital area without further notice); “death or suicide.” Same hospital readmissions and thus, the total count and the rate of and time to readmission within 1 year after discharge were included.

### Statistical Analysis

According to the principle of independence, the analysis only included the first admission between January 1st, 2013, and December 31st, 2019. For the analysis, we considered the day (i.e., weekdays and weekend days) and time (i.e., day, dawn, or dusk) of admission as the independent variable. Descriptive statistics (mean, SD, median, percentage) were used for demographic and clinical characteristics. Differences in categorical variables were assessed with the Chi-square Test, with a subsequent Chi-square omnibus comparison for the day of the week (i.e., weekdays vs. weekends) and time of the day (i.e., day vs. dawn vs. dusk) of admission (31). Using the residuals, we calculated the percentage a given category contributes to the Chi-squared score. Outcomes of interest were additionally represented as Odds Ratios (OR). Logistic regression was used to control confounders; we adjusted OR for age, sex, education, marital status, admission status, diagnosis, and severity. ANOVA assessed differences in continuous variables, with a pairwise comparison with the Student's *t*-test. If assumptions about the distribution were not met, we additionally used an alternative non-parametric test (i.e., the Kruskal–Wallis test and the Mann–Whitney *U*-Test). All tests of significance were two-tailed. Due to the large sample size, we chose a significance level of *p* < 0.01. However, considering that the day and time categories have a hierarchical structure, we decided to introduce a correction (by a factor of six); thus, we consider only *p*-values < 0.001 statistically significant. Effect sizes were assessed with eta-square for continuous and phi for categorical variables (32, 33). Kaplan Meier time-to-event curves were drawn and analyzed for the length of stay and time to readmission. To test for statistical significance, we used the log-rank *p*-value. Statistical analyses were conducted using the statistical software R (v4.0.3).

## Results

### Demographic and Clinical Characteristics of the Sample

During the study period, 12,449 direct patient admissions to an acute psychiatric ward were recorded. Most admissions (*n* = 10,452, 84.0%) occurred on a working day; the remaining (*n* = 1,997, 16.0%) were on a weekend or public holiday. Admissions occurred predominantly during daytime hours (*n* = 7,924, 63.7%). Correspondingly, admissions occurred during office hours (*n* = 6,886, 65.9%). The percentage on weekend days (*n* = 1,038, 52.0%) and outside office hours (*n* = 959, 48.0%) was similar.

The mean age of the sample was 48.05 ± 20.90 years, with 49.32% (*n* = 6,140) females. More than one-third of all admissions (39.7%, *n* = 4,950) were compulsory; the majority (41.0%, *n* = 2,030) of them was issued by a mental health professional (i.e., a psychiatrist); with almost equal proportions for emergency services (30.3%, *n* = 1,499) and non-mental health professionals (28.7%, *n* = 1,421). The mean CGI-S at admission scored 4.76 ± 1.10; the mean HoNOS sum score was 19.66 ± 7.42. The most frequent treatment diagnoses were: major depressive disorder (*n* = 3,232, 25.9%); anxiety and stress-related disorders (*n* = 2,300, 18.5%); and schizophrenia spectrum disorders (*n* = 2,226, 17.9%), which together accounted for two thirds of all admissions.

**Figure 1:**
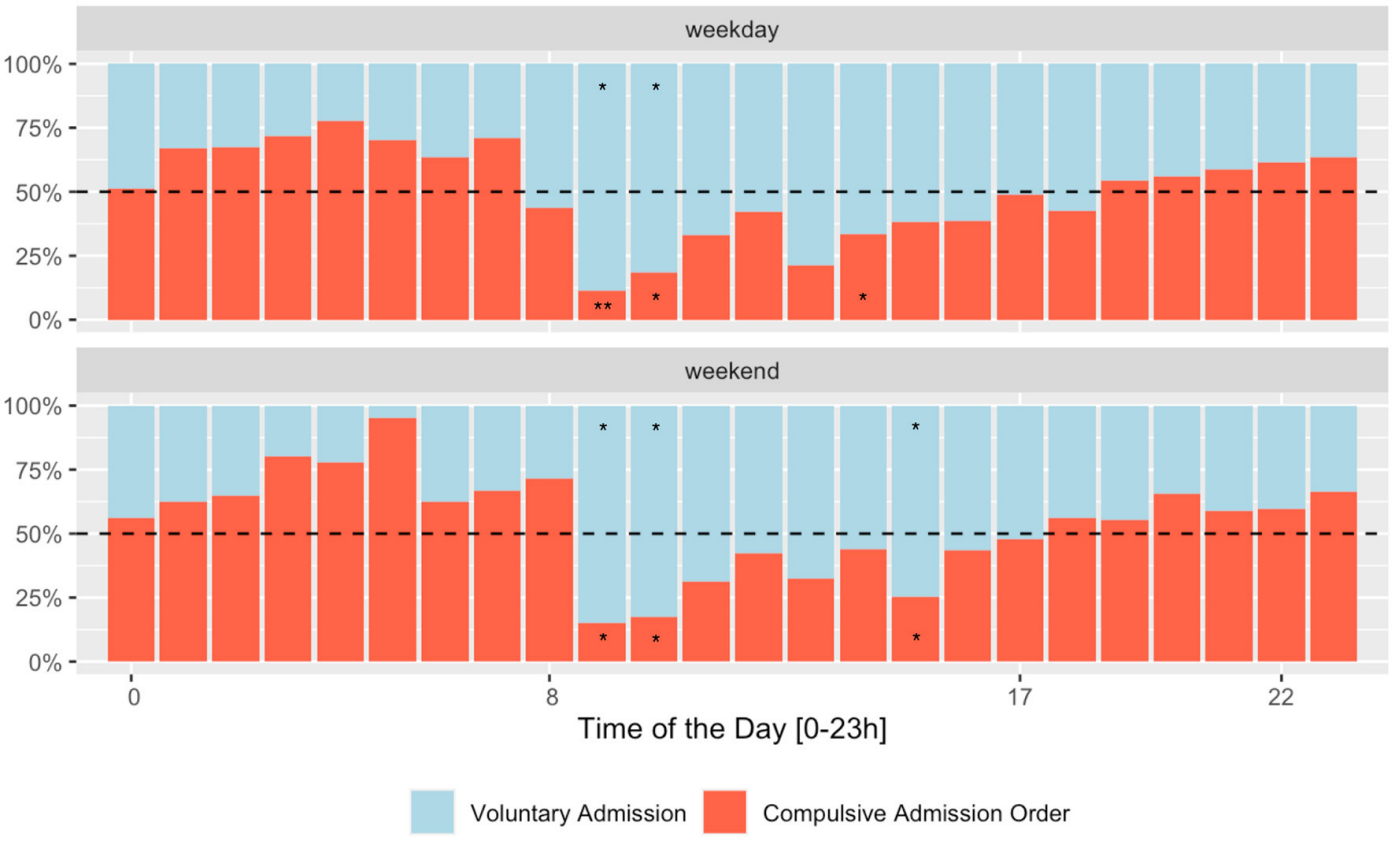
Rate of compulsory admission orders according to the day and time of admission. Category contributes *5–10%; **11–20%; and *** >20% of the total Chi-square score.

Patients admitted during the daytime on working days were slightly older. Females were more frequently admitted during the daytime (both working and weekend days) than their male counterparts. Swiss nationals were more likely to be admitted during office hours, while tourists and travelers were more frequently admitted at out-of-office hours. During office hours and daytime, fewer compulsory admissions were recorded: they steadily increased from dusk till dawn until a peak of compulsory admissions was reached around 5–6 a.m. The increase was more prominent on weekends (see Figure 1). The rate of voluntary admissions, especially self-referral, was higher during the daytime on weekends. Further demographic and clinical characteristics according to the day and time of admission are summarized in Table 1 and Figure 1.

**Table 1:** Demographic characteristics of the sample according to the day and time of admission.

	**Weekdays**			**Weekends and public holidays**					
	**Day**	**Dawn**	**Dusk**	**Day**	**Dawn**	**Dusk**	**Statistic**	***p*** **-value**	**Effect size**
	***N*** **= 6,886**	***N*** **= 581**	***N*** **= 2,985**	***N*** **= 1,038**	***N*** **= 236**	***N*** **= 723**			
	**M (SD)**	**M (SD)**	**M (SD)**	**M (SD)**	**M (SD)**	**M (SD)**			
Age	50.79 (21.56)^†^	41.34 (18.10)^††^	45.96 (20.50)^††^	43.66 (17.68)^††^	42.08 (18.05)	44.22 (19.05)	F_(5, 12, 443)_ = 60.88	<0.001	0.02
	***N*** **(%)**	***N*** **(%)**	***N*** **(%)**	***N*** **(%)**	***N*** **(%)**	***N*** **(%)**			
**Sex**							$X_{\left(1,12,449\right)}^{2}$ = 32.98	<0.001	0.05
Female	3,567 (51.8)^*^	251 (43.2)^**^	1,474 (49.4)	571 (55.0)^**^	111 (47.0)	335 (46.3)^*^	WD = WN; d ≠ dw; d ≠ du; dw = du		
Male	3,319 (48.2)^*^	330 (56.8)^**^	1,511 (50.6)	467 (45.0)^**^	125 (53.0)	388 (53.7)^*^	WD = WN; d ≠ dw; d ≠ du; dw = du		
**Marital Status**							$X_{\left(20,12,449\right)}^{2}$ = 165.28	<0.001	0.11
Single	3,233 (47.0)	320 (55.1)	1,518 (50.9)	531 (51.2)	109 (46.2)	372 (51.5)	WD = WN; d = dw; d ≠ du; dw = du		
Married	1,447 (21.0)	98 (16.9)	556 (18.6)	217 (20.9)	37 (15.7)	153 (21.2)	WD ≠ WN; d = dw; d = du; dw = du		
Separated/Divorced	1,230 (17.9)	77 (13.3)	485 (16.2)	158 (15.2)	45 (19.1)	107 (14.8)	WD = WN; d = dw; d = du; dw = du		
Widowed	555 (8.1)^**^	14 (2.4)^*^	193 (6.5)	37 (3.6)^*^	8 (3.4)	29 (4.0)	WD ≠ WN; d ≠ dw; d = du; dw ≠ du		
Other/Unknown	421 (6.1)^*^	72 (12.4)^**^	233 (7.8)	95 (9.2)	37 (15.7)^**^	62 (8.6)	WD ≠ WN; d ≠ dw; d = du; dw ≠ du		
**Education**							$X_{\left(15,12,449\right)}^{2}$ = 108.47	<0.001	0.09
Incomplete schooling	368 (5.3)	45 (7.7)	184 (6.2)	43 (4.1)	16 (6.8)	51 (7.1)	WD = WN; d = dw; d = du; dw = du		
Regular School	3,335 (48.4)^*^	308 (53.0)	1,678 (56.2)^**^	515 (49.6)	134 (56.8)	387 (53.5)	WD ≠ WN; d = dw; d ≠ du; dw = du		
Apprenticeship	2,096 (30.4)^***^	132 (22.7)	675 (22.6)^***^	283 (27.3)	53 (22.5)	175 (24.2)	WD = WN; d ≠ dw; d ≠ du; dw = du		
College/University	1,087 (15.8)	96 (16.5)	448 (15.0)	197 (19.0)^*^	33 (14.0)	110 (15.2)	WD = WN; d = dw; d = du; dw = du		
**Residency Status**							$X_{\left(15,12,449\right)}^{2}$ = 107.98	<0.001	0.09
Swiss Citizens	5,343 (77.6)	402 (69.2)	2,139 (71.7)	817 (78.7)	168 (71.2)	310 (67.4)	WD ≠ WN; d ≠ dw; d ≠ du; dw = du		
Residents	1,058 (15.4)	110 (18.9)	502 (16.8)	147 (14.2)	37 (15.7)	143 (19.8)^*^	WD = WN; d = dw; d = du; dw = du		
Refugee	116 (1.7)	11 (1.9)	89 (3.0)^**^	12 (1.2)	9 (3.8)	18 (2.5)	WD = WN; d = dw; d ≠ du; dw = du		
Tourists/travelers	369 (5.4)^**^	58 (10.0)^*^	255 (8.5)^**^	62 (6.0)	22 (9.3)	60 (8.3)	WD = WN; d ≠ dw; d ≠ du; dw = du		
**Language Proficiency**							$X_{\left(5,12,449\right)}^{2}$ = 46.97		
High German Proficiency	5,980 (86.8)	476 (81.9)	2,484 (83.2)	929 (89.5)	195 (82.6)	601 (83.1)	WD = WN; d ≠ dw; d ≠ du; dw = du		
Low German Proficiency	906 (13.2)^**^	105 (18.1)^**^	501 (16.8)^***^	109 (10.5)^***^	41 (17.4)	122 (16.9)	WD = WN; d ≠ dw; d ≠ du; dw = du		
**Help-seeking behavior**							$X_{\left(5,12,449\right)}^{2}$ = 1,009.7	<0.001	0.28
Compulsory Admission Order	2,006 (29.1)^**^	403 (69.4)^**^	1,606 (53.8)^**^	342 (32.9)	168 (71.2)^*^	425 (58.8)^*^	WD ≠ WN; d ≠ dw; d ≠ du; dw ≠ du		
Voluntary Admission	4,880 (70.9)^**^	178 (30.6)^*^	1,379 (46.2)^*^	696 (67.1)	68 (28.8)	298 (41.2)	WD ≠ WN; d ≠ dw; d ≠ du; dw ≠ du		
**Compulsory Admission Order**	*N* = 2,006	*N* = 403	*N* = 1,606	*N* = 342	*N* = 168	*N* = 425	$X_{\left(10,4,950\right)}^{2}$ = 37.34	<0.001	0.09
ED/Ambulance	658 (32.8)^**^	114 (28.3)	447 (27.8)^*^	117 (34.2)	47 (28.0)	116 (27.3)	WD = WN; d = dw; d ≠ du; dw = du		
Non-Mental health Professional	616 (30.7)^*^	103 (25.6)	444 (29.8)	94 (27.5)	50 (29.8)	114 (26.8)	WD = WN; d = dw; d = du; dw = du		
Mental Health Professional	732 (36.5)^***^	186 (46.2)^*^	715 (44.5)^**^	131 (38.3)	71 (42.3)	195 (45.9)^*^	WD = WN; d ≠ dw; d ≠ du; dw = du		
**Voluntary Admission**	*N* = 4,880	*N* = 178	*N* = 1,379	*N* = 696	*N* = 68	*N* = 298	$X_{\left(15,7,499\right)}^{2}$ = 133.97	<0.001	0.14
Self- Referral	2,655 (54.4)^*^	108 (60.7)	791 (57.4)	524 (75.3)^***^	37 (54.4)	187 (62.8)	WD ≠ WN; d ≠ dw; d ≠ du; dw ≠ du		
ED/Ambulance	693 (14.2)	30 (16.9)	222 (16.1)	55 (7.9)^**^	14 (20.6)	41 (13.8)	WD ≠ WN; d ≠ dw; d ≠ du; dw = du		
Non-Mental health Professional	577 (11.8)^*^	14 (7.9)	132 (9.6)	41 (5.9)^**^	5 (7.4)	17 (5.7)	WD ≠ WN; d ≠ dw; d ≠ du; dw = du		
Mental Health Professional	955 (19.6)	26 (14.6)	234 (17.0)	76 (10.9)^**^	12 (17.6)	53 (17.8)	WD ≠ WN; d ≠ dw; d ≠ du; dw = du		

*WD, weekday; WN, weekend; d, day; dw, dawn; du, dusk. Category contributes:*

* *5–10%;*

** *11–20%; and*

*** *>20% of the total Chi-square score. Pairwise comparisons:*

† *WD-d > all others;*

†† *WD-du > WN-d, WD-dw*.

**Figure 2:**
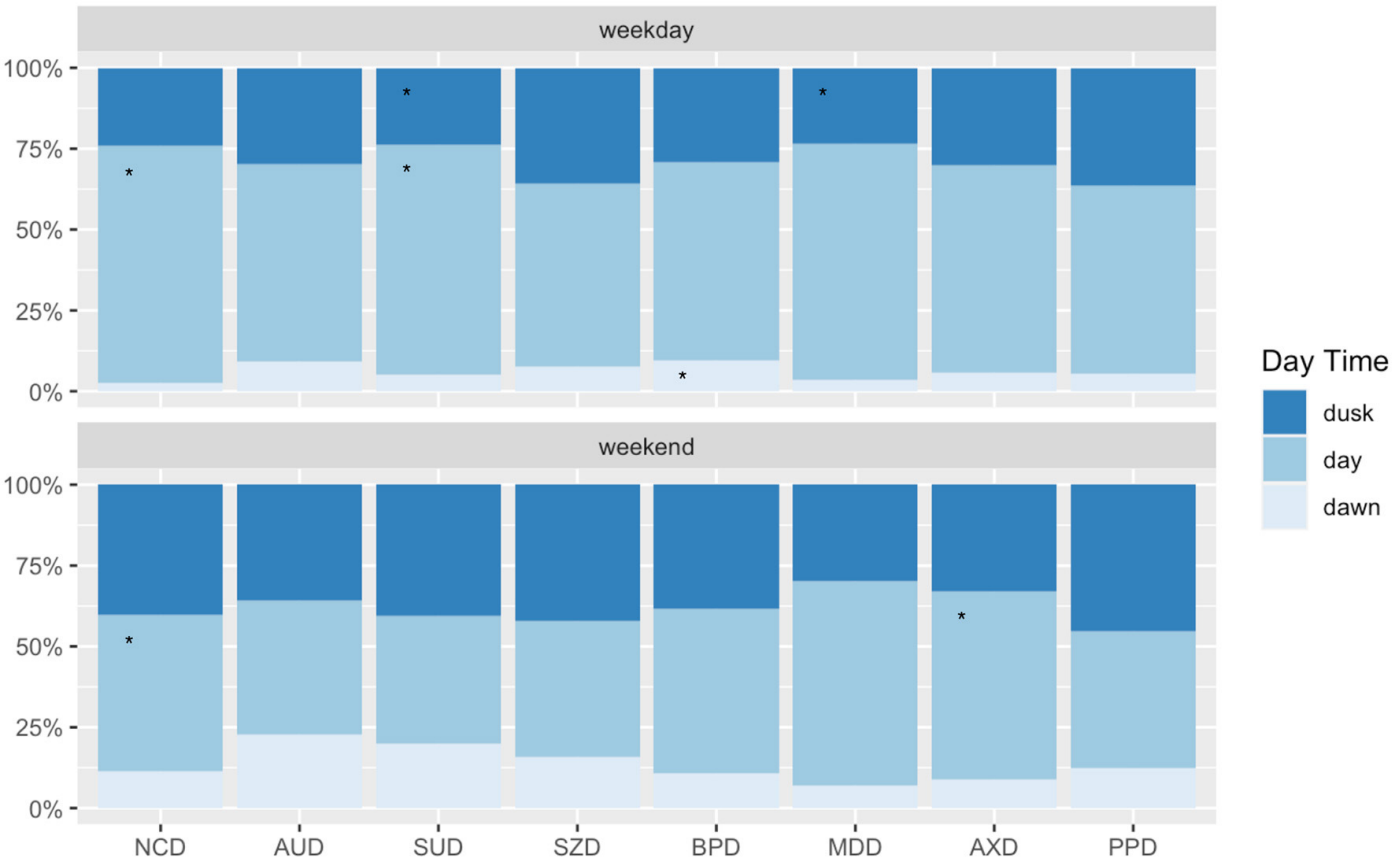
Main diagnosis according to the day and time of admission. NCD, Neurocognitive Disorders; AUD, Alcohol Use Disorders; SUD, Substance Use Disorders; SZD, Schizophrenia Spectrum Disorder; BPD, Mania and Bipolar Disorder; MDD, Major Depressive Disorder; SRD, Stress-Response Disorders; AXD, Anxiety Disorders; PPD, Personality Disorders; NDD, Neurodevelopmental Disorders. $X_{\left(45,9,943\right)}^{2}$ = 306.4; *p* <0.001. Category contributes *5–10%; **11–20%; and ***>20% of the total Chi-square score.

**Figure 3:**
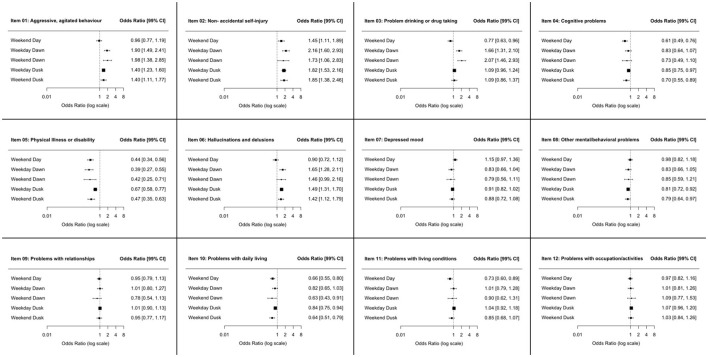
Odds Ratio (OR) and Confidence Intervals (CI) for the single Items of the Health of the Nation Outcome Scales (HoNOS) being rated three or four (i.e., clinically significant and part of the patients' care and treatment plan); patients admitted during the daytime of a weekday served as the reference group.

The admission rate for certain diagnoses varied from working days to weekends, increasing until dawn. This was the case of alcohol and substance use disorders, either as the main treatment diagnosis or comorbid disorder (See Figure 2). The clinical severity and burden showed a slightly differing profile according to the day and time of admission (see Figure 3); HoNOS Items rating “overactive, aggressive, disruptive or agitated behavior” (Item 01); “non-accidental self-injury” (Item 02) were higher after day time: “problem drinking or drug-taking” (Item 03) was higher at dawn irrespective of the day of the week; and “physical illness or disability problems” (Item 05) was higher at office hours. The CGI-S severity score and the HoNOS sum scores were lower on weekends, irrespective of the time of the day. However, the effect sizes are very small.

For further detail, see Table 2 and Figure 2.

**Table 2:** Clinical characteristics of the sample according to the day and time of admission.

	**Weekdays**			**Weekends and public holidays**					
	**Day**	**Dawn**	**Dusk**	**Day**	**Dawn**	**Dusk**	**Statistic**	***p*** **-value**	**Effect size**
	***N*** **= 6,886**	***N*** **= 581**	***N*** **= 2,985**	***N*** **= 1,038**	***N*** **= 236**	***N*** **= 723**			
	***N*** **(%)**	***N*** **(%)**	***N*** **(%)**	***N*** **(%)**	***N*** **(%)**	***N*** **(%)**			
**Diagnosis**									
							$X_{\left(40,12,449\right)}^{2}$ = 460.82	<0.001	0.180
Neurocognitive disorders	969 (14.1)^*^	35 (6.0)	321 (10.8)	63 (6.1)^*^	15 (6.4)	53 (7.3)	WD ≠ WN; d ≠ dw; d ≠ du; dw ≠ du		
Alcohol use disorder	449 (6.5)	67 (11.5)	220 (7.4)	66 (6.4)	36 (15.3)	57 (7.9)	WD ≠ WN; d ≠ dw; d = du; dw ≠ du		
Substance use disorders	464 (6.7)	32 (5.5)	154 (5.2)	34 (3.3)	17 (7.2)	35 (4.8)	WD ≠ WN; d = dw; d = du; dw = du		
Schizophrenia spectrum disorder	1,027 (14.9)^*^	140 (24.1)	652 (21.8)^*^	171 (16.5)	64 (27.1)	172 (23.8)	WD = WN; d ≠ dw; d ≠ du; dw = du		
Bipolar disorder	491 (7.1)	77 (13.3)^*^	235 (7.9)	75 (7.2)	16 (6.8)	57 (7.9)	WD = WN; d ≠ dw; d = du; dw = du		
Major depressive disorder	1,979 (28.7)	97 (16.7)	642 (21.5)^*^	325 (31.3)	35 (14.8)	154 (21.3)	WD = WN; d ≠ dw; d ≠ du; dw ≠ du		
Anxiety disorders	1,189 (17.3)	104 (17.9)	561 (18.8)	259 (25.0)^*^	40 (16.9)	147 (20.3)	WD ≠ WN; d = dw; d = du; dw = du		
Personality disorders	318 (4.6)	29 (5.0)	200 (6.7)	45 (4.3)	13 (5.5)	48 (6.6)	WD = WN; d = dw; d ≠ du; dw = du		
**Comorbid diagnosis**									
Comorbid substance use disorder	619 (9.0)	70 (12.0)^***^	259 (8.7)	81 (7.8)	40 (16.9)^***^	65 (9.0)	$X_{\left(5,12,449\right)}^{2}$ = 26.52	<0.001	0.040
*No comorbid substance use disorder*	6,267 (91.0)	511 (88.0)	2,726 (91.3)	957 (92.2)	196 (83.1)^*^	658 (91.0)	WD = WN; d ≠ dw; d = du; dw = du		
Comorbid personality disorder	506 (7.3)	55 (9.5)	222 (7.4)	81 (7.8)	24 (10.2)	56 (7.7)	$X_{\left(5,12,449\right)}^{2}$ = 5.92	0.31	-
*No comorbid personality* disorder	6,380 (92.7)	526 (90.5)	2,763 (92.6)	957 (92.2)	212 (89.8)	667 (92.3)	WD = WN; d = dw; d = du; dw = du		
	***M*** **(SD)**	***M*** **(SD)**	***M*** **(SD)**	***M*** **(SD)**	***M*** **(SD)**	***M*** **(SD)**			
**Admission**									
CGI- S	4.79 (1.07)^†^	4.87 (1.04)^††^/^†^^*††*^	4.74 (1.15)^††^	4.59 (1.08)^†/††^	4.72 (1.20)	4.66 (1.08)^†^/^†^^*††*^	F_(5, 12, 443)_ = 9.01	<0.001	0.004
HoNOS sum score	19.77 (7.33)^*f*^	20.16 (7.59)^††^	19.79 (7.59)^††^	18.43 (7.50)^††^/^*f*^	19.85 (7.27)	19.27 (7.31)	F_(5, 12, 443)_ = 7.13	<0.001	0.003
HoNOS rated ≥ 3	3.99 (2.39)^*f*^	4.09 (2.47)	4.01 (2.38)	3.59 (2.34) ^††^/^*f*^	3.97 (2.29)	3.77 (2.34)	F_(5, 12, 443)_ = 6.58	<0.001	0.003
**Discharge**									
CGI-I	2.61 (1.00)^*ff*^	2.46 (0.92)^††^/^*ff*^	2.52 (0.97)^*ff*^	2.61 (0.94)^††^	2.49 (0.97)	2.51 (0.96)	F_(5, 12, 443)_ = 6.47	<0.001	0.003
HoNOS sum score	10.89 (8.20)^*fff*^	10.14 (7.76)	10.06 (7.72)	8.05 (7.15)	9.02 (7.22)	9.27 (7.46)	F_(5, 12, 443)_ = 28.73	<0.001	0.009
HoNOS Items ≥ 3	1.50 (2.25)^*fff*^	1.27 (2.08)	1.28 (2.07)	0.85 (1.85)	1.06 (1.94)	1.04 (1.91)	F_(5, 12, 443)_ = 23.13	<0.001	0.009
**Outcomes**									
HoNOS sum score difference	8.79 (7.86)^*fff*^	10.02 (8.61)	9.73 (8.33)	10.39 (8.01)	10.83 (8.72)	10.00 (7.68)	F_(5, 12, 443)_ = 9.4	<0.001	0.004
HoNOS percentage of change	48.2 (38.1)^*fff*^	51.2 (43.7)	50.7 (35.8)	58.3 (31.9)	56.6 (34.7)	53.9 (31.6)	F_(5, 11, 374)_ = 6.7	<0.001	0.003
**Coercive measures**									
Compulsory retention	94 (1.4)	8 (1.4)	68 (2.3)	9 (0.9)	3 (1.3)	6 (0.8)	$X_{\left(5,12,449\right)}^{2}$ = 17.7	0.003	-
*No compulsory retention*	6,604 (98.6)	563 (98.6)	2,874 (97.7)	1,008 (99.1)	230 (98.7)	711 (99.2)	WD = WN; d = dw; d = du; dw = du		
Forced medication	213 (3.1)^***^	36 (6.2)^*^	170 (5.7)^***^	29 (2.8)^*^	19 (8.1)^**^	54 (7.5)^***^	$X_{\left(5,12,449\right)}^{2}$ = 76.5	<0.001	0.07
*No forced medication*	6,673 (96.9)	545 (93.8)	2,815 (94.3)	1,009 (97.2)	217 (91.9)	669 (92.5)	WD = WN; d ≠ dw; d ≠ du; dw = du		
Isolation- restrain	242 (3.5)^***^	53 (9.1)^***^	197 (6.6)^**^	32 (3.1)^*^	22 (9.3)^*^	60 (8.3)^**^	$X_{\left(5,12,449\right)}^{2}$ = 104.9	<0.001	0.09
*No isolation- restraint*	6,644 (96.5)	528 (90.9)	2,788 (93.4)	1,006 (96.9)	214 (90.7)	663 (91.7)	WD = WN; d ≠ dw; d ≠ du; dw = du		
**Service use parameters**									
	**MN (IQR)**	**MN (IQR)**	**MN (IQR)**	**MN (IQR)**	**MN (IQR)**	**MN (IQR)**			
Length of stay (days)	16 (33)^*fff*^	9 (22)	12 (28)	4 (15)	5 (21)	8 (27)	H_(5, 12, 449)_ = 278.1	<0.001	0.01
	***M*** **(SD)**	***M*** **(SD)**	***M*** **(SD)**	***M*** **(SD)**	***M*** **(SD)**	***M*** **(SD)**			
Length of stay (days)	24.70 (27.49)^*fff*^	18.74 (25.47)	22.80 (31.31)	15.52 (21.49)	15.50 (21.14)	20.19 (26.43)	F_(5, 12, 443)_ = 27.75	<0.001	0.01
	***N*** **(%)**	***N*** **(%)**	***N*** **(%)**	***N*** **(%)**	***N*** **(%)**	***N*** **(%)**			
Type of discharge							$X_{\left(10,12,449\right)}^{2}$ = 32.41	0.002	0.05
Regular	6,619 (96.1)	551 (94.8)	2,821 (94.5)	1,002 (96.5)	218 (92.4)	686 (94.9)	WD ≠ WN; d ≠ dw; d ≠ du; dw ≠ du		
Irregular	228 (3.3)	29 (5.0)	151 (5.1)	33 (3.2)	18 (7.6)	35 (4.8)	WD ≠ WN; d ≠ dw; d ≠ du; dw ≠ du		
Death/suicide	39 (0.6)	1 (0.2)	13 (0.4)	3 (0.3)	0 (0.0)	2 (0.3)	WD ≠ WN; d ≠ dw; d ≠ du; dw ≠ du		
Readmission	2,539 (36.9)	206 (35.5)	1,102 (36.9)	394 (38.0)	79 (33.5)	305 (42.2)	$X_{\left(5,12,449\right)}^{2}$ = 10.46	0.06	–
*No readmission*	4,347 (63.1)	374 (64.5)	1,883 (63.1)	644 (62.0)	157 (66.5)	418 (57.8)	WD ≠ WN; d ≠ dw; d ≠ du; dw ≠ du		
	***M*** **(SD)**	***M*** **(SD)**	***M*** **(SD)**	***M*** **(SD)**	***M*** **(SD)**	***M*** **(SD)**			
Number of readmissions	1.43 (0.94)	1.42 (0.85)	1.45 (1.00)	1.46 (1.01)	1.31 (0.72)	1.51 (1.04)	F_(5, 12, 442)_ = 12.05	0.07	–
Time to readmission (days)	99.63 (102.70)	111.05 (103.52)	102.27 (103.52)	102.92 (105.92)	91.46 (111.88)	102.48 (105.06)	F_(5, 3, 126)_ = 0.55	0.79	–

*WD, weekday; WN, weekend; d, day; dw, dawn; du, dusk. Category contributes*

* *5–10%;*

** *11–20%; and*

*** *>20% of the total Chi-square score. Pairwise comparisons:*

† *WD-d > WN-dw, WN-du;*

†† *WN-d > WD-dw, WD-du; ††† WD-dw > WN-du;*

f *WD-d > WN-d;*

ff *WD-d > WD-dw, WD-du;*

fff *WD-d > all others*.

### Service Use, Treatment, Outcomes According to the Day and Time of Admission

**Figure 4:**
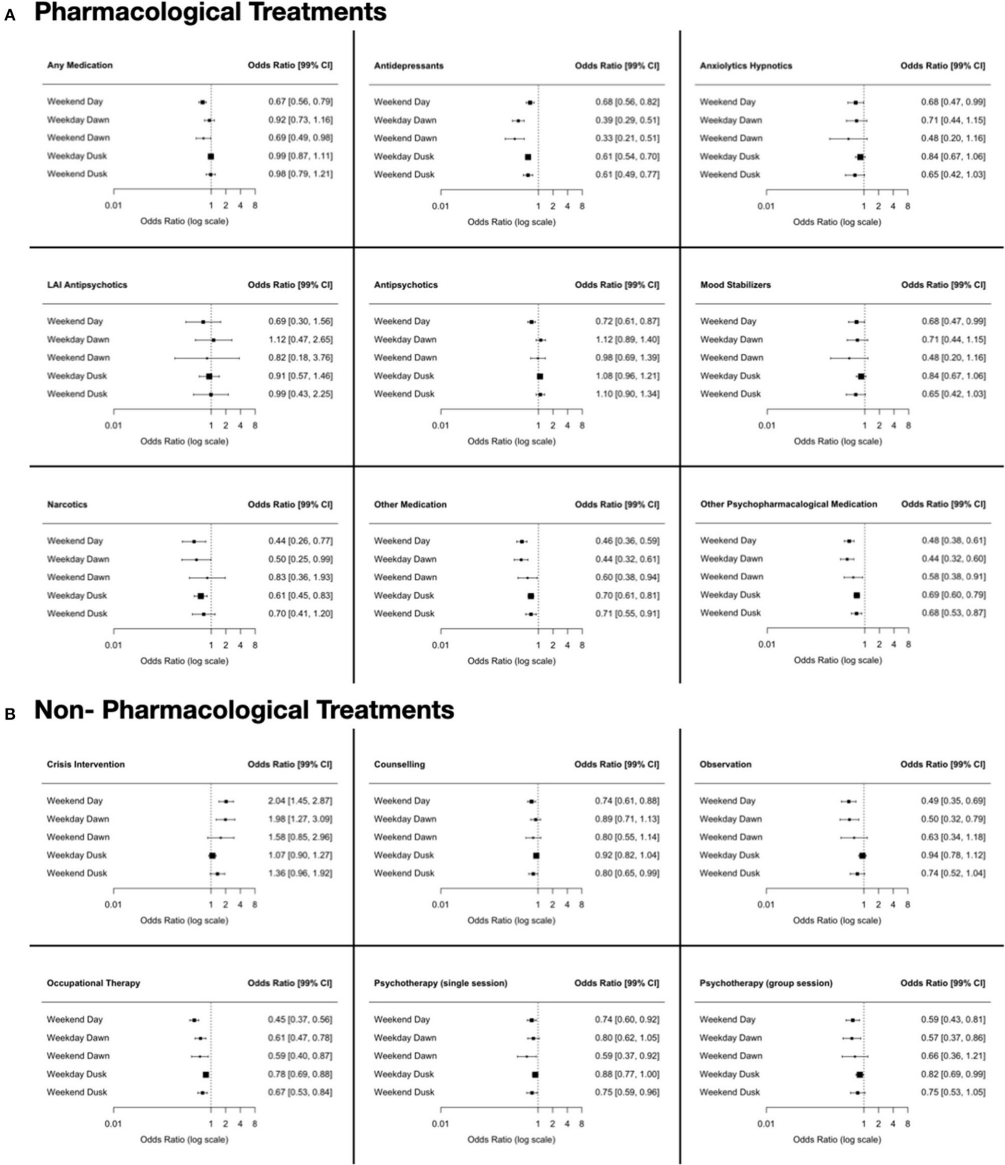
Odds Ratio (OR) and Confidence Intervals (CI) of treatment prescribed according to the day and time of admission; patients admitted during the daytime of a weekday served as the reference group. **(A)** Pharmacological Treatment. **(B)** Non-Pharmacological Treatment.

**Figure 5:**
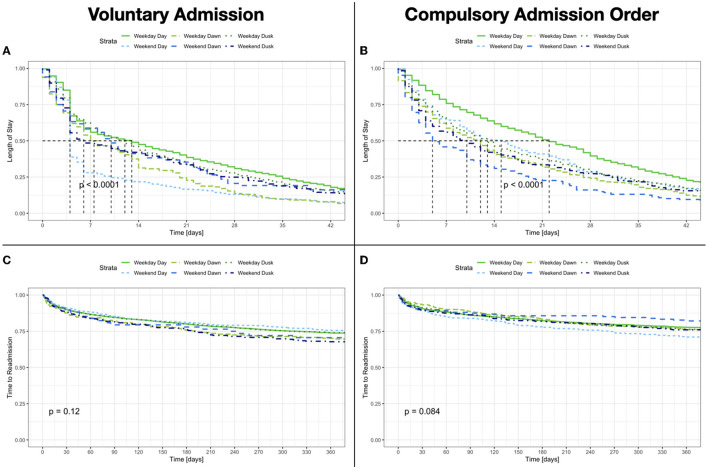
**(A,B)** Length of Stay according to the day and time of admission and admission status. **(C,D)** Time to readmission according to the day and time of admission and admission status.

There are slight differences regarding pharmacological and non-pharmacological treatment perceived according to the day and time of admission. Patients admitted outside of office hours generally had fewer prescribed therapies (See Figures 4A,B). These differences disappeared after correcting for confounders (age, sex, education, marital status, admission status, diagnosis, and severity). The mean length of stay was 20.62 ± 28.52 days with a right-skewed distribution and a median of 13 (IQR: 29) days. Patients admitted during the daytime of a weekday had a longer length of stay than all others (24.70 ± 27.49). Compulsorily admitted patients almost doubled the length of stay of those voluntarily admitted [*F*_(5, 12, 429)_ = 4.539, *p* < 0.001]; although this difference remained significant only for those admitted during daytime (See Table 2, Figures 5A,B). Patients voluntarily admitted during the daytime on the weekend had a shorter length of stay with a median of 4 days. The rate of compulsory retention was similar among all groups. The rate of and time to readmission and the total count of subsequent readmissions were similar for all groups (For further details, see Table 2, Figures 5C,D).

**Figure 6:**
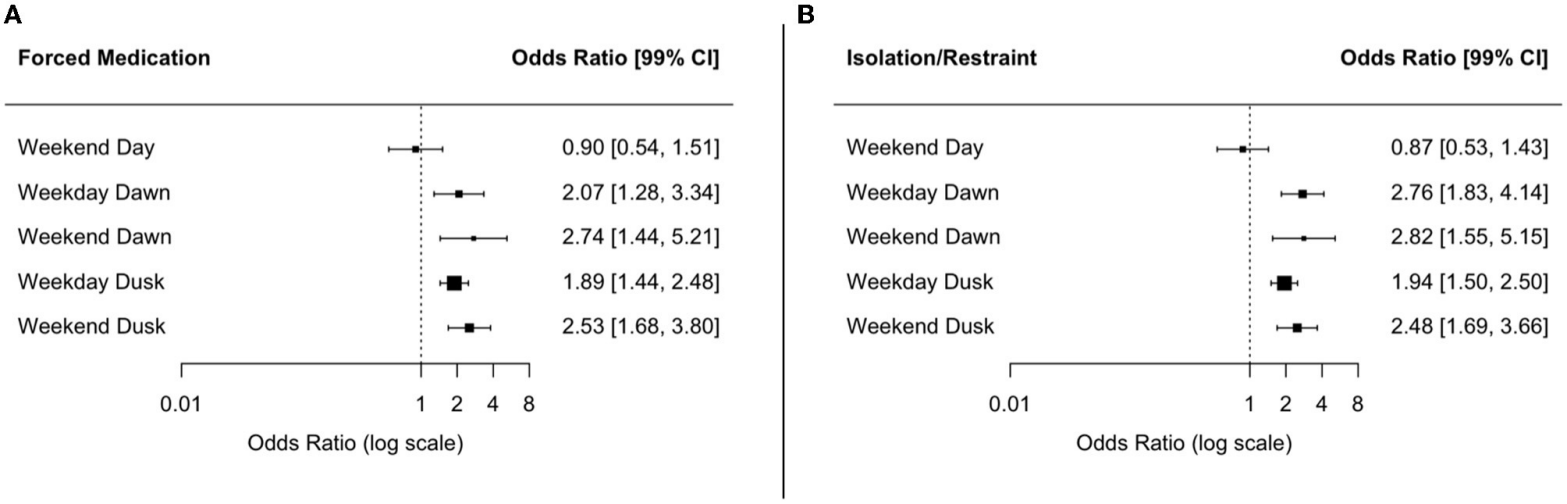
Odds Ratio (OR) and Confidence Intervals (CI) of coercive measures according to the day and time of admission; patients admitted during the daytime of a weekday served as the reference group. **(A)** Forced Medication. **(B)** Isolation Restraint.

Clinical ratings showed a significant improvement, regardless of the day and time of admission. The CGI-I ratings showed a robust clinical improvement, so did the HoNOS sum score difference among all groups between admission and discharge [*F*_(11, 22, 610)_ = 960.05, *p* < 0.001; η^2^ = 0.30]. Nonetheless, patients admitted outside office-hours (i.e., all except daytime on working days) achieved a significantly higher sum score difference (office hours 9.05 ± 8.04 vs. cumulative outside office-hours 8.11 ± 7.60; *F*_(5, 12, 443)_ = 9.4, *p* < 0.001) and correspondingly a higher percentage (office hours 46.3 ± 38.1 vs. cumulative outside office hours 42.6 ± 40.7; *F*_(5, 11, 374)_ = 6.7, *p* < 0.001) of improvement (see Table 2). However, the effect size of the difference was small. Thus, the total count of clinically relevant domains could be reduced during hospitalization. The differences in the clinical ratings between the day and time of admission were also statistically significant but again showed an overall very low effect size (see Table 2). Patients admitted at evening and night shifts showed higher rates of coercive measures (i.e., forceful medication and seclusion/restrain) irrespectively of the day of the week (see Figures 6A,B).

## Discussion

We show that the day and time of admission are related to help-seeking behavior and the pathway to admission. Patients admitted outside the daytime (either on weekdays or weekends) might have shared characteristics, leading to an involuntary admission through a compulsory admission order. While hospitalized, the rate of coercive measures for these patients was correspondingly higher. Overall, they also showed a shorter length of stay, with a higher rate of irregular discharge. Nevertheless, a large proportion of patients involuntarily admitted were willing to remain voluntarily in psychiatric treatment. The background for these findings is complex; and reflects the interrelationships between legislation, mental health services, and individual diagnosis and psychopathology.

Psychiatric disorders often show a chronic course of disease with varying severity, intercalated with episodic exacerbations (34–36). In contrast to other medical disciplines, compulsory admission is intended and legally regulated for people who have a mental condition. Therefore, treatment can be imposed on subjects who are either unaware of a disorder or unwilling to get treatment without their consent (17, 37), thus potentially leading to compulsory admission orders during episodes of acute exacerbations. A compulsory admission order can be issued when a person exhibits a debilitating mental condition (i.e., a psychiatric disorder) and when less restrictive means of care and treatment are not feasible. Nevertheless, few alternatives to psychiatric hospitalization outside daytime hours are available. The strikingly higher rate of compulsory admission in late and night shifts might be a combination of several factors, where the interplay between legislation, mental health services, and the patients' clinical characteristics play a pivotal role (38–40).

Previous studies consistently report a relationship between compulsory admissions and out of office hours (6, 37, 41, 42), noting that this association changes in relation to global legal and mental health service peculiarities. For example, previous reports from Great Britain suggest lower counts of compulsory admission on weekends, where two independent physicians are required to issue a compulsory admission order (5, 43). In contrast, our results and several previous reports from Switzerland and Germany show higher rates of compulsory admissions, where just one physician is required for issuing a compulsory admission order. In Switzerland, the patient may appeal against the compulsory admission order. In Germany, compulsory admission has to be confirmed by a judge within the first 72 h (37, 42). The difference between Great Britain, on the one hand, and Germany and Switzerland, on the other hand, is the legal threshold for compulsory admission when alternatives for crisis intervention are rare during out-of-office hours. The differences in rates of compulsory admission orders might result from differences in community mental health resources (17).

Patients admitted outside business hours generally received fewer interventions, especially counseling, psychotherapy, and medication. In line with previous findings, the differences in treatment showed no real influence on the clinical outcome (5, 44). Overall, patients showed a robust clinical improvement. Minimal differences related to the day and time of admission were detected, resulting in only small effect sizes. Patients admitted outside daytime hours experienced a shorter stay (6) and thus achieved improvement in less time. The remaining service use parameters, especially the rate of and time to readmission, were identical.

Patients compulsorily admitted had a length of stay nearly twice as long as those voluntarily admitted, although this difference remained only significant for daytime admissions on weekdays. Patients voluntarily admitted during the daytime of weekends had a shorter length of stay with a median of 4 days. Previous studies from Switzerland have shown a shorter length of stay for compulsory admissions (45, 46). Others from Italy have shown a longer length of stay (47, 48). However, these analyses did not take the diagnosis and clinical characteristics of the patients fully into account. Beyond patient characteristics, as diagnosis and clinical profile, these results can be explained by differences in legislation and mental health service availability. We could interpret these admissions as in-hospital crisis interventions due to the lack of alternatives on the weekend (38, 39, 49).

When comparing our results with those from other medical disciplines, we have to consider several peculiarities of psychiatry. First, in psychiatric crises and emergencies, death, loss of physical integrity, or disability are usually not expected outcomes (50). Second, the admission process in psychiatry is an intervention on its own, capable of changing the course of the disease and influencing outcomes (49). However, it remains unclear if admission to outpatient treatment, either compulsory or voluntary, outside daytime hours is sufficient to relieve an acute crisis or emergency or lead to an admission to inpatient treatment (51, 52). Third, patients admitted outside daytime hours feature a different demographic and clinical profile, leading to compulsory admission. Therefore, differences in treatment (and subsequent outcomes) can partly be attributed to these characteristics. This contrasts to other medical specialties, where differences in treatment and outcome could be mainly attributed to a service organization and staff availability. Finally, we must acknowledge that the goal of most hospitalizations is not symptomatic remission or recovery; it is aimed to pave the way for further outpatient treatment (53, 54). The readmission rates and time to readmission reflect the effects of an outpatient treatment capable of reducing the readmission rate and cumulative length of stay (55, 56).

The main strength of our study is the large clinical sample collected under “real-life” conditions. It allows robust statistical analysis and generalization of our results. One potential source of bias is that we limited the sample to patients admitted to the general psychiatric ward. This limitation is justifiable because these wards are responsible for the basic psychiatric treatment in the catchment area and provide care for upcoming admissions round the clock. Thus, the same hospital readmission presence is a surrogate for relapse. These wards also have facilities and personnel capacities sufficient to execute compulsory interventions safely and provide services comparable to primary mental health systems in other countries. We limited the analysis to the first admission in the observation period. Thus, we cannot rule out previous hospitalizations of a given individual patient; we consider that the large observation period can mitigate the influence of these cases. We defined “help-seeking behavior” according to the type of admission (i.e., voluntary or involuntary); and the pathway to admission (i.e., referral); this allows us to infer how the patients tried to get help (57). Nonetheless, we have no direct patient-reported measures regarding the reasons and preferences for seeking psychiatric consultation or treatment.

From our results, we can conclude that day and time of admission are to some extent related to help-seeking behavior, the pathway to admission, as well as to the severity of psychiatric illness. Moreover, it is related to a shorter length of stay and higher risk of coercive measures, with similar rates of treatment prescribed and clinical outcomes. Accordingly, we assume a close interplay between legislation and service availability here. These results should encourage the implementation of outpatient mental health services, available from dusk till dawn.

## Data Availability Statement

The data supporting the findings of this study are available from the corresponding author upon reasonable request. The data are not publicly available due to privacy or ethical restrictions.

## Ethics Statement

The studies involving human participants were reviewed and approved by Ethics Committee of the Canton of Zurich. Written informed consent for participation was not required for this study in accordance with the national legislation and the institutional requirements.

## Author Contributions

ES, BQ, SV, and SE: contributed to conception and design of the study. SE: organized the database and performed the statistical analysis. MK and DM: wrote the first draft of the manuscript. MK, AH, VN, KR, and FF: wrote sections of the manuscript. All authors contributed to manuscript revision, read, and approved the submitted version.

## Conflict of Interest

The authors declare that the research was conducted in the absence of any commercial or financial relationships that could be construed as a potential conflict of interest.

## Publisher's Note

All claims expressed in this article are solely those of the authors and do not necessarily represent those of their affiliated organizations, or those of the publisher, the editors and the reviewers. Any product that may be evaluated in this article, or claim that may be made by its manufacturer, is not guaranteed or endorsed by the publisher.
